# Autoimmune B Cell Repertoire in a Mouse Model of Sjögren’s Syndrome

**DOI:** 10.3389/fimmu.2021.666545

**Published:** 2021-04-23

**Authors:** Manuel Sáez Moya, Rebeca Gutiérrez-Cózar, Joan Puñet-Ortiz, María Luisa Rodríguez de la Concepción, Julià Blanco, Jorge Carrillo, Pablo Engel

**Affiliations:** ^1^ Immunology Unit, Department of Biomedical Sciences, Medical School, University of Barcelona, Barcelona, Spain; ^2^ IrsiCaixa AIDS Research Institute, Badalona, Spain, Germans Trias i Pujol Research Institute (IGTP), Catalonia, Spain; ^3^ AIDS and Related Diseases Chair, Universitat de Vic-Central de Catalunya (UVIC-UCC), Vic, Spain; ^4^ August Pi i Sunyer Biomedical Research Institute (IDIBAPS), Barcelona, Spain

**Keywords:** B cell repertoire, autoantibodies, polyreactive antibodies, Sjögren’s syndrome, monoclonal antibodies

## Abstract

In genetically prone individuals, chronic immune activation may lead to expansion of autoreactive lymphocyte clones that can induce organ damage developing autoimmune disorders. Sjögren’s Syndrome (SjS) is a systemic chronic autoimmune disease that primarily affects exocrine glands. Despite the accumulated evidences of profound B-cell alterations of humoral immunity, the repertoire and development of B-cell autoreactivity in SjS remains to be determined. We hypothesize that SjS mice will have an increased frequency of self-reactive B cells with a progressive evolution to antigen-driven oligoclonality. Here, we study the B cell repertoire of NOD.H-2^h4^ mice, a mouse model of spontaneous autoimmunity mimicking SjS without developing diabetes. A library of 168 hybridomas from NOD.H-2^h4^ mice and 186 C57BL/6J splenocytes at different ages was created. The presence of mono or polyreactive autoantibodies to several antigens was evaluated by ELISA, and their staining patterns and cellular reactivity were tested by IFA and FACS. We observed a higher frequency of autoreactivity among B-cell clones from NOD.H-2^h4^ mice as compared to wild-type mice. The presence of polyreactive and autoreactive IgG clones increased with mice age. Strikingly, all anti-Ro52 autoantibodies were polyreactive. No loss of polyreactivity was observed upon antibody class switching to IgG. There was a progression to oligoclonality in IgG B cells with mice aging. Our results indicate that in the NOD.H-2^h4^ mouse model of SjS, IgG+ B cells are mainly polyreactive and might expand following an unknown antigen-driven positive selection process.

## Introduction

Sjögren’s syndrome (SjS) is a chronic autoimmune disease, of unknown etiology, that primarily affects the salivary and lacrimal glands with progressive dryness of mouth and eyes (1, 2). SjS is one of the most common systemic autoimmune diseases (3). One of the hallmarks of SjS is inflammation of the exocrine tissue, termed focal lymphocytic sialadenitis, and the presence of anti-nuclear antibodies such as anti-dsDNA and anti-Ro52 that are useful diagnostic markers (4–6). Importantly, these autoantibodies have been shown to play a pathogenic role (4, 7). Besides the occurrence of these autoantibodies, SjS patients are characterized by deep alterations in the frequency of several B lymphocyte populations, both in the blood and in the infiltrated exocrine glands (8–10). Another hallmark of the disease is the B cell hyperreactivity due to chronic antigenic stimulation, which is initially polyclonal but can progress to monoclonal B cell lymphoproliferation. Ultimately, leading to B cell lymphoma development, being the most common marginal zone B-cell lymphoma (11, 12).

SjS patients have increased susceptibility to suffer other autoimmune diseases such as rheumatoid arthritis or systemic lupus erythematosus (13). There is accumulated evidence that in susceptible individuals, chronic B-cell activation may lead to the expansion of autoreactive lymphocytes that can lead to organ damage developing autoimmune disorders (14, 15). This is due to defective B cell tolerance checkpoints that are characteristic of systemic autoimmune diseases. This deficient negative selection leads to the increase in the generation of polyreactive antibodies that are characterized by their ability to bind to multiple structurally unrelated antigens including autoantigens (16). These antibodies are also present in non-autoimmune prone individuals, representing an important part of immune repertoires under physiological conditions and may play essential roles in immune defense and in the maintenance of immune homeostasis (17, 18). B cells producing these antibodies have been postulated to provide the immune system with primordial specificities that upon antigen recognition can be induced to generate highly antigen-specific antibodies (19). Importantly, the presence of polyreactive antibodies has been associated with different autoimmune and inflammatory processes (20–22). A high prevalence of naïve B cells expressing polyreactive antibodies has been reported in patients with systemic lupus erythematosus and rheumatoid arthritis (23). Thus, a physiological balance between useful protective and detrimental polyreactive antibodies has to be established in order to avoid detrimental effects.

Despite the evidence of profound B cell disturbances, there is poor understanding of the underlying mechanisms involved in development and evolution of B cell autoreactivity in SjS. We hypothesized that the B cells of aged SjS mice will have an increased frequency of self-reactive B cells reflecting impaired tolerance checkpoints. Besides, under chronic antigen exposure, B cell clones expressing polyreactive or autoreactive IgM antibodies might switch to IgG and tune their specificity, becoming high affinity monospecific. Moreover, we predicted that B cells of the SjS mice may evolve to oligoclonality, translated as the use of progressively more restricted V gene segments (24). In order to test these hypotheses, we analyzed the B cell repertoire of NOD.H-2^h4^, which spontaneously develops SjS, presenting exocrine glands infiltrations (25). Similar to SjS patients, these mice present exocrine gland disease, but in contrast to NOD mice, they do not develop diabetes (26). In addition, these mice develop autoantibodies to antinuclear antigens preceding the development of lymphocytic infiltration in the salivary glands (26). We generated a large library of hybridomas and tested the reactivity of the secreted monoclonal antibodies and determined the gene usage of heavy and light chains variable genes segments.

Our results showed elevated frequency of polyreactive B cell clones in SjS mice that increased with age. Strikingly, all anti-Ro52 autoantibodies were polyreactive. There was a progression to oligoclonality of IgG B cells without loss of polyreactivity. Our results indicate that IgG+ autoreactive B cells are mainly polyreactive and might expand following an unknown antigen-driven positive selection process.

## Material and Methods

### Mice

NOD.H-2^h4^ (NOD.Cg-H2^h4^/DilTacUmm), NSG (NOD Scid Gamma) and C57BL/6J mice were purchased from Jackson Laboratory and breed under specific pathogen free conditions at the animal house facility from Faculty of Medicine, University of Barcelona. Mice experiments were performed according to the European Community Directive 2010/63/EU and Spanish legislation (Real Decreto 53/2013, BOE-A-2013-101337) regulating the protection and usage of laboratory animals. Experimental procedures were approved by the Ethics Committee for Animal Experiments (CEEA) of the University of Barcelona.

### Hybridoma Generation

Generation of hybridomas was done using all splenocytes derived from unimmunized NOD.H-2^h4^ (28, 47 and 66 w.o.) and C57BL/6J (26, 47 and 69 w.o.) mice. One animal and two animals of each age were used for the generation of hybridomas from the NOD.H-2^h4^ and C57BL/6J mice respectively. The fusions of splenocytes of C57BL/6J mice correspond to pools of two animals in order to increase the number of hybridomas. In contrast, in NOD.H-2^h4^ mice no splenocytes pools were done, due to the high number of hybridomas obtained in each fusion. Splenocytes were fused to mouse myeloma NS1 cells following standard fusion protocols as described before (27). Briefly, splenocytes were isolated by manual disaggregation under sterile conditions and fused with NS1 cells using polyethylene glycol (Sigma). The fused cells were plated into 96-well plates in selective medium containing RPMI-1640 (Sigma) with 0’5% Hybridoma Fusion and Cloning Supplement (Roche), Hypoxanthine-Aminopterin-Thymidine (HAT) (Sigma), 20% of FBS-Good Australian Origin (DDBiolab), L-glutamine (Gibco) and Penicillin-Streptomycin (Gibco). Hybridomas were incubated at 37°C in 5% CO_2_ conditions for 7-10 days. Hybridoma supernatants were tested by ELISA to confirm antibody production; IgM clones were saved when high antibody concentration and only one clone was observed on the 96 well plate, all IgG clones were saved except those which were co-expressing IgM in the same well, and IgGs were subcloned.

### Immunoglobulin Quantification and Isotyping

Quantification of immunoglobulin and isotyping were done by ELISA; coating 96-well microtiter plates (Costar) with 3 µg/mL of F(ab’)₂ Fragment Goat Anti-Mouse IgM, µ chain specific (Jackson ImmunoResearch) or Goat anti-mouse IgG (Sigma) polyclonal antibodies. Hybridoma supernatants were diluted 1/2 for the determination of antibody production; and 1/20 for IgG subclass determination. Production and isotype determination were tested with Biotin Rat anti-mouse IgM (R6-60.2, BD Biosciences) and Horseradish Peroxidase (HRP)-conjugated goat anti-mouse IgG (Sigma). IgG subclasses determination was done using Biotin anti-mouse IgG1, IgG2b, IgG2c and IgG3 polyclonal antibodies (Jackson ImmunoResearch). HRP-conjugated streptavidin (Roche) was used for biotinylated antibodies detection. Finally, microplates were developed with TMB (BD Bioscience) and reaction was stopped with 2M H_2_SO_4_. Microplates were read with an EPOCH Microplate Spectrophotometer (BioTek) at 450-570 nm.

### Flow Cytometry

Supernatants of highly confluent hybridoma cultures were diluted 1/2 and tested by flow cytometry against mouse L cells which were previously fixed (4% PBS-formaldehyde) and permeabilized (0’05% PBS-Triton X-100). FITC Goat anti-mouse IgM (Southern Biotech) or PE F(ab’)₂ Fragment Goat Anti-Mouse IgG, Fcγ fragment specific (Jackson ImmunoResearch) were used as polyclonal detection antibodies. Data was acquired with FACSCalibur flow cytometer (BD Biosciences) and FlowJo vX.0.7 (Tree Star, Inc) software was used to analyze the results. Flow cytometry experiments were performed as described before (28).

### Antibody Reactivity by ELISA

Reactivity determination was done using high binding plates (Costar) coated with the following antigens at 3 μg/mL: calf thymus double-stranded DNA (Sigma); Histone (H1; Sigma); Ro52 (SSA-TRIM21; Sigma); LPS from Escherichia coli O55:B5 (Sigma); human Insulin (Lilly) and Ovalbumin (InvivoGen). Supernatants of highly confluent hybridoma cultures were diluted 1/2; HRP-conjugated goat anti-mouse IgG (Sigma) polyclonal antibody and Biotin rat anti-mouse IgM (R6-60.2, BD Biosciences) were used as detection antibodies and HRP-conjugated streptavidin (Roche) for the biotinylated antibody detection. Finally, microplates were developed with TMB (BD Bioscience) and reaction was stopped with 2M H_2_SO_4_. Microplates were read with an EPOCH Microplate Spectrophotometer (BioTek) at 450-570 nm. Antibodies which were positive for three or more antigens of our panel were considered polyreactive.

### Immunofluorescence Assay

Supernatants of highly confluent hybridoma cultures were analyzed by IFA using HEp-2 cells which were fixed (4% PBS-formaldehyde) and permeabilized (0’05% PBS-Triton X-100); and cryosections from kidney, liver, external lacrimal and submandibular salivary glands obtained from NSG mice, fixed and permeabilized (Acetone); both blocked (PBS with 6% FBS). Then, cells and cryosections were incubated with 1/2 diluted supernatants. Anti-mouse IgG Fc Alexa488 (Life Technologies) and anti-mouse IgM Fc FITC (Southern Biotech) were used as polyclonal detection antibodies. Finally, both immunocytochemistry and immunohistochemistry slides were prepared using coverslips and aqueous mounting medium with anti-fading: 90 mL Glycerol (VWR), 10 mL PBS 1X, 500 mg Propyl-gallate (Sigma). Sections were visualized at 20X and 40x magnifications using NIKON e600 microscope.

### Immunoglobulin VH and VL Sequencing

Hybridoma cells were harvested by centrifugation and stored at -80 °C as dry pellets until use. Total RNA was purified from those dry pellets using the RNeasy Mini Kit (Qiagen). Total RNA was reverse transcribed in a final volume of 20 µL/sample and using the SuperScript III Reverse Transcriptase (Invitrogen). Mouse IgH and IgK V gene transcripts were amplified independently with a battery of selected sense and antisense primers (**Supplementary Tables 1**, **2**) (29). All PCR reactions were performed in a final volume of 25 µL/sample and using the Platinum Taq DNA Polymerase High Fidelity (ThermoFisher). PCR products were analyzed on 2% agarose gels and the correct amplifications were purified using the Zymoclean Gel DNA Recovery (ZymoResearch) and send to sequencing (Macrogen Europe, Madrid, Spain).

### IGHV and IGKV Rearranged Sequences Analysis

IGHV and IGKV sequences were analyzed first aligning all obtained sequences with the Sequencher v5.4.6 (GeneCodes, Corp.) software and all aligned sequences then were analyzed with the mouse immunoglobulin repertoire set of the IMGT/V-QUEST reference directory (30, 31). Only productive sequences were used for the study, and V(D)J genes including CDR1, CDR2 and CDR3 were deeply analyzed for the IGHV and IGKV. Hybridomas sharing the same IGHV and IGKV genes and nucleotide/amino acid mutations were considered as B-cell clones, and those who shared IGHV and IGKV but had additional mutations were considered clonally related.

### Antibody Purification

Hybridoma antibodies that were selected for assessing their avidity were purified using the Affi-Gel Protein A MAPS II Kit (BioRad) and following the manufacturer’s protocol.

### Antigen Reactivity Strength Assay

Selected antibodies were analyzed for their antigen reactivity strength by ELISA. High binding plates (Costar) were coated with the previously mentioned panel of antigens at 3 μg/mL. Serial dilutions (range 100 μg/mL - 0’01 μg/mL) of purified antibodies were tested in duplicate. HRP-conjugated goat anti-mouse IgG (Sigma) was used as detection polyclonal antibody. Finally, microplates were developed with TMB (BD Bioscience) and reaction was stopped with 2M H_2_SO_4_. Microplates were read with an EPOCH Microplate Spectrophotometer (BioTek) at 450-570 nm.

### Statistical Analysis

P-values for antibody autoreactivity, analysis of antibody reactivity against Ro52 and antibody reactivity were calculated by χ^2^ test, P-values for VH amino acid changes were calculated by unpaired t test.

## Results

To determine the B cell repertoire of SjS-like mice, we generated a library of 168 hybridomas from 28, 47 and 66 weeks old female NOD.H-2^h4^ mice. It has been described that all 28-week old female NOD.H-2^h4^ mice shows evidence of salivary gland lymphocyte infiltration and the presence of anti-Ro52 and anti-dsDNA autoantibodies (26). Additionally, we also generated a collection of 186 hybridomas from female C57BL/6J (B6) mice (ages 26, 47 and 69 weeks).

### Elevated Frequency of Autoreactive B Cell Antibodies in Aged NOD.H-2^h4^ Mice

**Figure 1:**
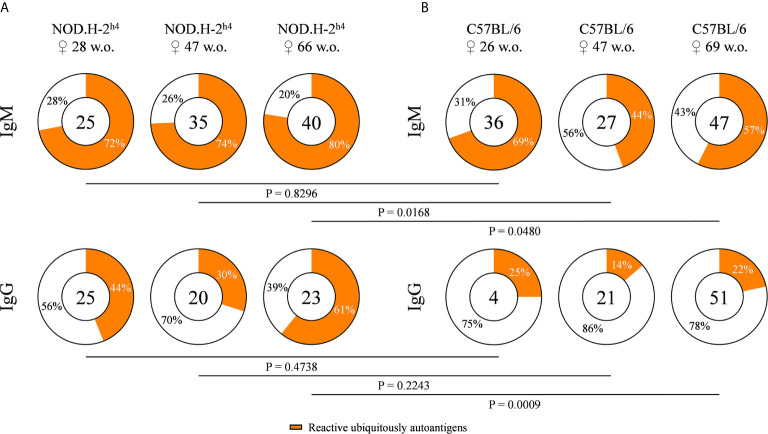
Frequency of autoreactive antibodies derived from NOD.H-2^h4^ and C57BL/6J mice. Pie charts showing the proportion of hybridomas that produce autoreactive (orange) or non-autoreactive (white) antibodies determined by flow cytometry with murine L cells. Data from NOD.H-2^h4^ (age: 28, 47 and 66 w.o.) **(A)**, and C57BL/6J (age: 26, 47 and 69 w.o.) **(B)** and both IgM and IgG isotypes are shown. The number of tested antibodies is indicated in the pie chart center. Data are pooled from independent experiments for each mouse age. P-values were calculated by χ^2^ test.

In order to evaluate the frequency of antibodies against ubiquitous self-antigens, we tested the reactivity of the supernatants of our hybridoma collection with permeabilized cells of the mouse fibroblast cell line L. The frequency of IgM and IgG autoreactive antibodies was significantly increased in autoantibodies from SjS mice as compared to B6 wild-type mice, especially in mice with an age over 60 weeks (**Figures 1A, B** **)**. A significant increase in the frequency of autoreactive IgG with age was observed in SjS mice, reaching a 61% of the IgG antibodies tested in 66 old mice, revealing defects in B cell tolerance in these mice. In contrast, the frequency of IgM autoreactive antibodies did not increase with age (**Figure 1A**). These data show that autoreactive clones dominate the B cell repertoire of NOD.H-2^h4^ mice.

### High Frequency of Autoreactive B Cell Antibodies Recognized Ro52 in SjS Mice

**Figure 2:**
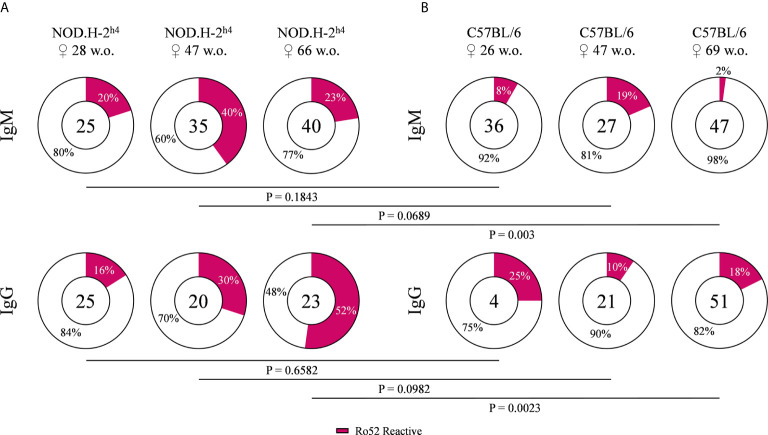
Frequency of Ro52 specific antibodies. Pie charts showing the proportion of Ro52 reactive (magenta) and non-reactive (white) antibodies, from NOD.H-2^h4^ **(A)** and C57BL/6J **(B)**, determined by ELISA. Data for each age NOD.H-2^h4^: 28, 47 and 66 w.o., C57BL/6J: 26, 47 and 69 w.o.; and both IgM and IgG isotypes are shown. The number of tested antibodies is indicated in the pie chart center. Data are pooled from independent experiments for each mouse age. P-values were calculated by χ^2^ test.

We determined the frequency of anti-Ro52 autoantibodies, since Ro52 is a characteristic target of anti-nuclear autoantibodies in patients with SjS (4–6). B cells from old SjS mice (over 40 weeks) presented a higher frequency of antibodies reactive with Ro52 as compared with B6 mice of both IgM and IgG. The percentage of anti-Ro52 IgG antibodies in the SjS mice increased dramatically with age to the point that more than 50% of the B cell clones were reactive with this antigen in 66-week old animals (**Figures 2A, B** **)**. Surprisingly, we also detected a very high percentage of anti-dsDNA antibodies in these animals (**Supplementary Figure 1**). These data showed that most of the autoantibodies could be reactive with both antigens at the same time, indicating a high percentage of polyreactive antibodies.

### Most of the Autoreactive B Cell Antibodies Present Polyreactivity

**Figure 3:**
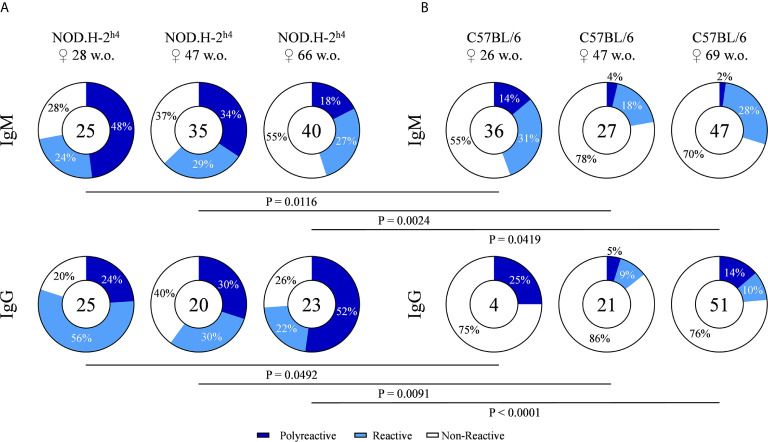
Frequency of polyreactive antibodies derived from NOD.H-2^h4^ and C57BL/6J mice. Antibody reactivity against dsDNA, H1, Ro52, LPS, Insulin and OVA was evaluated by ELISA. Pie charts showing the proportion of polyreactive (reactivity ≥ 3 antigens) (dark blue), reactive (one or two antigen) (light blue) and non-reactive (white) antibodies determined by ELISA. Data for each age NOD.H-2^h4^: 28, 47 and 66 w.o. **(A)**; C57BL/6J: 26, 47 and 69 w.o. **(B)**] and both IgM and IgG isotypes are shown. The number of tested antibodies is indicated in the pie chart center. Data are pooled from independent experiments for each mouse age. P-values were calculated by χ^2^ test.

In order to explore the levels of polyreactivity, the monoclonal antibodies of our hybridoma collection were tested for their reactivity with a panel of six structurally unrelated molecules that included double strand DNA (dsDNA), histone 1 (H1), Ro52, lipopolysaccharide (LPS), insulin (INS) and ovalbumin (OVA). Antibodies reactive with three or more of these antigens were considered as polyreactive. The results shown in **Figure 3** demonstrate a higher frequency of polyreactivity in SjS mice as compared with B6 mice. These differences were observed for both IgM and IgG antibodies at all tested ages (**Figures 3A, B** **)**. Moreover, in the SjS mice the fraction of polyreactive antibodies increased with age, reaching more than 50% in 66-week old mice. These results demonstrate that most of the autoreactivity corresponds to polyreactive antibodies in SjS old mice.

### NOD.H-2^h4^ Polyreactive IgGs Increase Their Antigen Binding Reactivity With Aging

**Figure 4:**
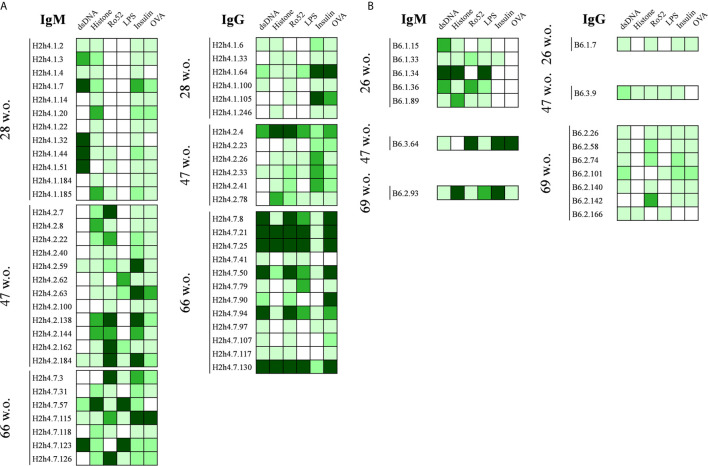
Polyreactivity profile of antibodies derived from NOD.H-2^h4^ and C57BL/6J mice. Heat maps showing the antibody binding to a panel of selected antigens (dsDNA, H1, Ro52, LPS, Insulin and OVA). Data of hybridomas derived from NOD.H-2^h4^ **(A)** and C57BL/6J **(B)** are shown. Color intensity is proportional to the reactivity level measured by ELISA, with darker green indicating high binding and light green showing moderate and low binding (white: no binding).

A semiquantitative reactivity analysis of the polyreactive antibodies is presented in **Figures 4A, B**. Although most antibodies presented distinct patterns of polyreactivity, an increase in the antigen binding reactivity of the IgG antibodies from aged SjS mice was detected. In contrast, this increase in antibody strength was not observed within the IgM antibodies from SjS mice and IgG antibodies from B6 mice. In addition, one of our most relevant observations of this study was that all antibodies reactive with Ro52 were actually polyreactive (**Figure 4A**).

### Analysis of V(D)J Sequences of the Polyreactive Antibodies Indicates High Level of Oligoclonality in SjS Mice

The genes encoding the variable regions of both the heavy and the light chain of the polyreactive antibodies were sequenced. We observed that polyreactive IgM and IgG antibodies both in NOD.H-2^h4^ and B6 mice used a very heterogeneous repertoire of variable gene segments (**Tables 1**, **2** and **Supplementary Tables 3**, **4**). The heavy chain CDR3 presented a length of 7 to 15 amino acids that has been reported to be a standard length for IgM antibodies (32). Most of the IgM antibodies presented nearly germ line encoded sequences (less than 5 mutations, data not shown).

**Table 1:** IGHV and IGKV of IgM antibodies derived from aging NOD.H-2^h4^ mice (28, 47 and 66 w.o).

	Hybridoma ID	Heavy Chain					Light Chain			
		IGHV	IGHD	IGHJ	CDR3	Length	IGκV	IGκJ	CDR3	Length
**28 w.o.**	**H2h4.1.2**	1-22	2-4	3	ARDDYDEAWFAY	12	3-10	2	QQNNEDPYT	9
	**H2h4.1.3**	3-1	3-3	1	ARGDRWYFDV	10	10-94	2	QQYSKLYT	8
	**H2h4.1.4**	14-2	1-1	4	ARDYGSSGYYYAMDY	15	13-85	2	QQYWGTPPT	9
	**H2h4.1.7**	5-4	1-1	1	ARVLRYFDV	9	12-44	2	QHHYGTPYT	9
	**H2h4.1.14**	14-3	2-10	2	ARSGYGNNDYFDY	13	4-74	5	QQYHSDPVT	9
	**H2h4.1.20**	1-39	2-12	3	ARGPYYSESGGFAY	14	6-20	2	GQSYSYPYT	9
	**H2h4.1.22**	1-43	3-1	2	ARYPRPV	7	3-4	2	QQSKEFPYT	9
	**H2h4.1.32**	5-6	4-1	2	ARVFEANFYFDY	12	12-46	1	QHFYGTPWT	9
	**H2h4.1.44**	5-2	4-1	2	AANWERLT	8	1-133	1	LQATHFPRT	9
	**H2h4.1.51**	10-3	4-1	3	VGLSGTAY	8	5-45	4	QQSNSWPFT	9
	**H2h4.1.184**	9-1	3-3	3	VKRGFAY	7	1-133	2	VQGTHFPYT	9
	**H2h4.1.185**	5-6	1-1	2	ARHNYGSALFDY	12	3-10	1	QQNNEDPWT	9
**47 w.o.**	**H2h4.2.7**	1-80	4-1	3	APNWAWFAY	9	1-122	2	LQVTHVPYT	9
	**H2h4.2.8**	1-39	–	4	ARSSMDY	7	6-15	5	EQYSSYPLT	9
	**H2h4.2.22**	1-5	1-1	2	ARVGEGY	7	2-109	5	AQMLERPLT	9
	**H2h4.2.40**	1-64	2-13	2	AKGGSTRGDY	10	4-78	5	QQYSGYLT	8
	**H2h4.2.59**	8-8	1-1	2	ARIGGSKNFDY	11	12-40	2	QHYYSTPYT	9
	**H2h4.2.62**	2-2	4-1	2	ARKGPTGYYFDY	12	4-81	2	QQWSGYPYT	9
	**H2h4.2.63**	2-2	4-1	2	ARKGPTGYYFDY	12	6-b	1	QQHYSSPWT	9
	**H2h4.2.100**	1-43	2-1	2	ARSTMVTTYYFDY	13	1-110	1	FQGTHVPWT	9
	**H2h4.2.138**				NS		10-96	1	QQDSKHPPT	9
	**H2h4.2.144**	1-15	2-2	3	TRDYGFAYW	9	12-46	5	QHYYSTPLT	9
	**H2h4.2.162**	1-69	2-5	4	ARRSNYDAMDY	11	1-133	2	VQGTHFPHT	9
	**H2h4.2.184**	1-63	1-1	2	ARDGYYGSSYDY	12	2-109	1	AQMLERPRT	9
**66 w.o.**	**H2H4.7.3**	1-26	3-3	2	ARSLYYFDY	9	NS			
	**H2H4.7.31**	1-12	1-3	2	ARSRWNWYYFDY	12	19-93	5	LQYDNLPLT	9
	**H2H4.7.57**	1-78	2-12	2	ARLRRRGS	8	4-57-1	1	QQGWDYPPT	9
	**H2H4.7.115**	1-7	1-1	1	ARPSTVVDWYFDV	13	1-133	4	VQGTHFPFT	9
	**H2H4.7.118**	1-55	3-2	2	AREAGDFDY	9	4-53	2	QQWSSYPYT	9
	**H2H4.7.123**				NS		5-39	5	QNGHSFPLT	9
	**H2H4.7.126**	1-19	4-1	1	AKTGTWYFDV	10	2-137	5	MQSLEYPLT	9

IGHV, Immunoglobulin heavy chain variable region gene; IGHD, Immunoglobulin heavy chain diversity region gene; IGHJ, Immunoglobulin heavy chain joining region gene; IGκV, Immunoglobulin kappa chain variable region gene; IGκJ, Immunoglobulin kappa chain joining region gene.

**Table 2:** IGHV and IGKV of IgG antibodies derived from aging NOD.H-2^h4^ mice (28, 47 and 66 w.o).

	Hybridoma ID	IgG Subclass	Heavy Chain					Light Chain			
			IGHV	IGHD	IGHJ	CDR3	Length	IGκV	IGκJ	CDR3	Length
**28 w.o.**	**H2h4.1.6**	IgG2b	3-3	1-1	4	ARGIFSYYYGSSYEGGYAMDY	21	5-45	5	QQSNSWPLT	9
	**H2h4.1.33**	IgG2b	NS					4-59	2	YQWSSYPYT	9
	**H2h4.1.64**	IgG2b	1-7	1-1	4	ARGGGNYVIRDYAMDY	16	12-44	5	QHHYGTPLT	9
	**H2h4.1.100**	IgG2b	9-1	1-1	2	VDYYVSSYGY	10	5-45	5	QQSNSWPLT	9
	**H2h4.1.105**	IgG2b	1-82	1-1	3	ARGGSSFFAY	10	6-15	4	QQFSTSPFT	9
	**H2h4.1.246**	IgG2b	1-26	1-1	4	ARRGVTKGYYAMDY	14	6-15	1	QQYSSSPRT	9
**47 w.o.**	**H2h4.2.4**	IgG2b	2-5	3-1	2	ARTRRSGFFDY	11	12-40	2	QHYYSTPYT	9
	**H2h4.2.23**	IgG2b	10-3	6-3	4	VREAGLYAMDY	11	3-1	1	QQSRKVPWT	9
	**H2h4.2.26**	IgG1	2-5	3-1	2	ARTRRSGFFDY	11	12-44	1	QHYYSTPPT	9
	**H2h4.2.33**	IgG2b	2-9-1	1-1	4	ARNRGNLPHYYDLDY	15	6-20	5	GQSYSYPLT	9
	**H2h4.2.41**	IgG1	5-4	1-3	4	ALGGGCAMDY	10	4-50	5	QQFTSSPSIT	10
	**H2h4.2.78**	IgG1	5-6	4-1	2	ARPKTGAFDY	10	1-132	2	LQGTYYPHT	9
**66 w.o.**	**H2h4.7.8**	IgG2b	1-4	1-1	2	ARSGGGDYGSSLCY	14	4-91	2	QHGSSLLRT	9
	**H2h4.7.21**	IgG2c	5-6	2-4	1	ARQRTRLRRGVRGYFDV	17	12-46	5	QHYYSVLFT	9
	**H2h4.7.25**	IgG2c	5-6	2-4	1	ARQRTRLRRGVRGYFDV	17	12-46	5	QHYYSVLFT	9
	**H2h4.7.41**	IgG2c	1-9	2-12	2	AITHHFDY	8	9-129	4	LQYASFT	7
	**H2h4.7.50**	IgG2b	1-4	1-1	2	ARSGGGDYGSSLCY	14	4-91	2	QHGSSLLRT	9
	**H2h4.7.79**	IgG2b	1-85	3-1	2	ARRGSYYFDY	10	12-46	2	QHFYGTPYT	9
	**H2h4.7.90**	IgG2b	1-53	2-3	2	ARWLLGDY	8	11-125	1	LQHSYLPWT	9
	**H2h4.7.94**	IgG2b	1-4	1-1	2	ARSGGGDYGSSLCY	14	4-91	2	QHGSSLLRT	9
	**H2h4.7.97**	IgG2c	2-2	1-1	4	ARVDPYLYGSSYNYAMDY	18	10-94	1	QQYSKLPWT	9
	**H2h4.7.107**	IgG1	1-81	2-3	2	ARYQGYGDY	9	15-103	1	LQGQSYPWT	9
	**H2h4.7.117**	IgG2b	1-53	2-2	4	ARVNGYLYAMDY	12	12-46	1	QHFWGTWT	8
	**H2h4.7.130**	IgG2b	1-59	2-9	2	ARSPLLWLRRRYYFDY	16	12-44	5	QHHYGTPLT	9

IGHV, Immunoglobulin heavy chain variable region gene; IGHD, Immunoglobulin heavy chain diversity region gene; IGHJ, Immunoglobulin heavy chain joining region gene; IGκV, Immunoglobulin kappa chain variable region gene; IGκJ, Immunoglobulin kappa chain joining region gene. Clonally related clones shade in blue and orange colors.

**Figure 5:**
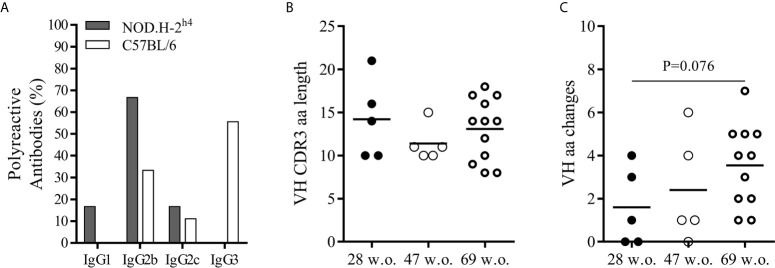
Mutational analysis and IgG subclass differences between polyreactive antibodies of NOD.H-2^h4^ and C57BL/6J. Frequency of polyreactive IgG subclasses in NOD.H-2^h4^ and C57BL/6J mice **(A)**. Data are pooled from independent experiments for NOD.H-2^h4^ and C57BL/6J mice. CDRH3 amino acid length **(B)** and number of VH replacing mutations **(C)** are shown. Data are pooled from independent experiments for each NOD.H-2^h4^ and C57BL/6J mice age. P-values were calculated by unpaired t test.

The vast majority of the polyreactive IgG antibodies were of the IgG2b subclass, whereas most of the polyreactive antibodies from the B6 mice corresponded to the IgG3 (**Figure 5A**). Polyreactive IgG antibodies presented heavy chain CDR3 ranging from 8 to 21 amino acid, although the great majority had a size of 7 to 15 amino acids. No significant difference was observed in the length of CDR3 of the variable region of the heavy chain of the SjS mice with age (**Figure 5B**). Interestingly, we observed an increase trend in the number of non-synonymous mutations affecting amino acids in the heavy chain variable region of antibodies with age in SjS mice (**Figure 5C**). This is a relevant observation suggesting antigen driven positive selection.

In 66-week old SjS mice, we observed two sets of identical sequences corresponding to IgG antibodies (**Table 2**). Antibodies H2h4.7.21 and H2h4.7.25 presented the same variable gene segment usage and sequence. These two antibodies presented multiple R amino acids in their heavy chain CDR3 which are highly correlated with polyreactivity. In addition, antibodies H2h4.7.8, and H2h4.7.50 shared the same sequence, while H2h4.7.94 uses the same variable gene segments both in the heavy and light chain. However, it presented three and two extra mutations within the heavy and light chain variable regions respectively, as compared with the other two clones. Mutations in the heavy chain variable region were located in the FR2 but near the CDR2, in the center of the CDR2 and in the center of the FR3. Whereas, mutations in the light chain were located in the center of the FR1 and in the CDR1. The majority of mutations within the heavy and light chain variable regions were dissimilar amino acid changes so a positive selection may have been taking part of (**Supplementary Table 5**). These data show that 66-week old mice presented a high level of oligoclonality.

### H2h4.7.94 Antibody Increases Its Antigen Reactivity Strength Without Losing Its Polyreactivity Due to Somatic Mutation

**Figure 6:**
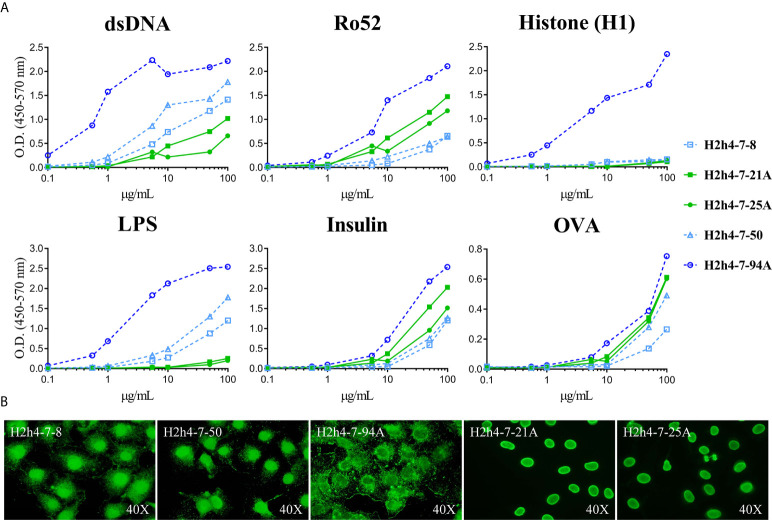
Antigen reactivity strength of polyreactive antibodies. The antigen reactivity strength of polyreactive antibodies (H2h4-7-8, H2h4-7-21A, H2h4-7-25A, H2h4-7-50 and H2h4-7-94A) to dsDNA, H1, Ro52, LPS, Insulin and OVA was determined by ELISA (ranging from 100 μg/mL - 0’01μg/mL) **(A)**. Antibodies sharing the same IGHV and IGKV sequences are represented with the same color (blue or green); H2h4-7-94A represented in dark blue has additional mutations compared to H2h4-7-8 and H2h4-7-50 represented in light blue. Representative images of immunocytochemistry with HEp-2 cells showing the cellular patterns of each antibody **(B)**.

Antigen reactivity strength of the five polyreactive antibodies that correspond to the two expanded clones was tested at several concentrations with the set of antigens. As expected, the antibodies with the same sequence presented identical reactivity patterns (**Figure 6A**). In contrast, antibody H2h4.7.94 presented an important increase of antigen reactivity strength to all the antigens recognized as compared with the two clonally related antibodies (H2h4.7.8 and H2h4.7.50) as shown by its high binding at low antibody concentrations. These antibodies were also tested using HEp-2 cells and they presented identical staining patterns within antibodies that were clonally related (**Figure 6B**). Although the staining pattern was the same between clonally related hybridomas, H2h4.7.94 presented a stronger cytoplasmic staining. These data show that antigen driven somatic mutation is able to increase the affinity of autoreactive clones maintaining the levels of polyreactivity.

## Discussion

Autoantibodies, especially against nuclear antigens, are the hallmark of several systemic autoimmune diseases, such as SjS. These antibodies arise due to the dysregulation of central and peripheral tolerance checkpoints (33, 34). Our analysis of the B cell repertoire of NOD.H-2^h4^ mice, which spontaneously develops a SjS-like disease, show that these mice presented higher frequencies of autoreactive B cell clones, against ubiquitous antigens, as compared to B6 mice that are relatively resistant to develop autoimmunity. Autoreactive IgM levels in SjS mice were modestly higher than in B6 mice. However, this difference became significantly higher in older mice, particularly for autoreactive IgGs in SjS mice, compared to B6 autoreactive IgG levels. The surprising frequency of autoreactive clones is identical to that reported by others (35, 36). In fact, more than 60% of the antibodies analyzed in SjS 66-week old mice were autoreactive. These data are consistent with the profound alteration of the B cell repertoire observed in SjS patients (16).

In this study, we have found increased levels of polyreactive IgG antibodies in the NOD.H-2^h4^ mice that significantly increased with mice aging. Surprisingly, this IgG producing B cells did not lose their polyreactive capacity even when their isotype switched from IgM to IgG. These findings are in line with previous studies of polyreactivity levels in patients with SjS, SLE and other mouse models of autoimmune diseases such as MRL/lpr (37–39). These studies showed that not only total IgG antibody, but also polyreactive IgG antibodies, levels in serum were higher in patients with SjS and SLE compared to healthy individuals.

A very striking observation was that all polyreactive IgG antibodies in the SjS mice corresponded to IgG2b and IgG2c isotypes as compared to the B6 mice where IgG3 isotype was the most abundant. IgG2b and IgG2c are known to be isotypes more pathogenic than the IgG3 antibodies in SLE mouse models (40, 41). Interestingly, this has also been observed in BAFF transgenic (Tg) mice that develop an autoimmune phenotype associated with the production of IgG2c and IgG2b autoantibodies (42). Although BAFF-Tg mice develop germinal centers they can produce class-switched antibodies even in the absence of T cells, suggesting that the activation of autoreactive B cells in these mice may occur in extrafollicular localizations. BAFF-Tg mice develop autoantibodies, leading to salivary gland destruction, features that are reminiscent of SjS. Moreover, in these mice, sialadenitis correlates with the aberrant accumulation of MZ-like B cells in the salivary glands a phenomenon also observed in the NOD.H-2^h4^ mice (43). Remarkably, MZ B cells are known to produce autoantibodies and to be a large source of polyreactive antibodies (44). It has been described that the number of splenic MZ B cells are increased in NOD (parental strain of NOD.H-2^h4^) autoimmune mice (45–48). We have also observed a dramatic increase in the number of MZ B cells with age in NOD.H-2^h4^ mice (data not shown). Unfortunately, a limitation of our study is that we have used whole spleen to produce monoclonal antibodies, thus we do not know the specific cellular origin of the hybridomas. There is further evidence that immature B T1 cells can produce isotype class-switch to IgG2b and IgG2c in a T-independent TLR7 dependent way (49, 50). Thus, it has been suggested that a combination of increased levels of immature cells and TLR7 dysregulation could thus predispose immature B cells to produce autoantibodies (51). More recently, it has been shown that TLR9 ligand CpG substantially boosted IgG2b and IgG2c antibody responses to virus-like particles in TCR-KO mice (52). All these observations indicate that the autoantibody production that we observed in our mouse model may be related to a dysfunction of the early T1 checkpoint.

In our study we have found higher frequencies of anti-Ro52 autoantibodies (IgM and IgG) in the NOD.H-2^h4^ mice, while in the B6 mice these autoantibodies were relatively uncommon. Antibodies directed against Ro52 are key biomarkers included in the current diagnostic criteria and likely involved in disease progression. These autoantibodies are present in the majority of SjS patients (60-70%) and their presence has been postulated to be linked with a bad prognostic of disease progression (53–55). Moreover, we observed that anti-Ro52 IgG antibodies increased significantly with mice aging in SjS mice as compared with B6 mice.

Strikingly, we found that all anti-Ro52 autoantibodies were polyreactive. This surprising result is consistent with the observation, described in a recent study that shows that anti-Ro52 antibodies from SjS patients presented high levels of polyreactivity (14). The role of polyreactive antibodies against autoantigens directly linked to autoimmune diseases has already been reported by other research groups for SLE. It has been observed that anti-DNA antibodies in both mouse models and SLE patients, cross-react with other autoantigens such as the phospholipid, cardiolipin or other ribonucleoproteins as Sm; then, those antibodies are considered polyreactive. These anti-DNA polyreactive antibodies have been associated with the disease pathology (56). Whereas monospecific anti-DNA antibodies did not generate lupus nephritis, polyreactive anti-DNA antibodies did. These findings indicated that polyreactivity is a pathologic characteristic for lupus nephritis (57–59). Moreover, another study demonstrates that anti-DNA/anti-NMDAR (N-methyl-D-aspartate receptor) cross-reactive antibodies generate damage in the hippocampus and also caused a flexible memory deficit, when penetrating the blood brain barrier (60). These events found in SLE due to polyreactive anti-DNA antibodies could also be relevant in other autoimmune diseases such as SjS and directly affecting the salivary and lacrimal glands.

The Ig gene sequencing of the polyreactive antibodies did not show any difference on variable gene segments usage between NOD.H-2^h4^ mice and B6 mice. These results differ from those reported in SjS patients, where public clonotypes or genetic polymorphisms are associated with the presence of autoreactive B cell clones. Al kindi et al. characterized public clonotypes such as the use of IGHV3-23 or IGHV3-7/IGK3-20 variable gene segments associated to anti-Ro52 antibodies between non-related SjS patients, other public clonotypes associated to other autoantigens such as La/SSB or Sm were also characterized (61). Although we did not find any evidence of this variable gene segment usage deviation or any differences in VH CDR3 length and charge, other studies also found a great diversity between SjS patient groups when analyzing variable gene segment usage and mutational differences (62). Additionally, it has been shown that even within the same patient when analyzing samples from different tissues the B cell repertoire is very different as is the case in multiple sclerosis (MS) when comparing B cells from peripheral blood and cerebrospinal fluid (63).

Furthermore, we identified two sets of clonally related IgG hybridomas (one set of 2 IgG2c and another one of 3 IgG2b), using the same variable gene segments and presenting the same nucleotide mutation pattern. Moreover, in the set of 3 clonally related IgG hybridomas one of them showed more mutations than the other two, indicating additional rounds of antigen driven SHM. Surprisingly, the polyreactive antibody that acquired amino acid changes increased its antigen reactivity strength towards autoantigens without losing its polyreactivity capacity. These 2 sets of clonally related B cells correspond to more than 20% of the total IgG obtained and analyzed from the 66-week old NOD.H-2^h4^ mice, so this B cell clonal expansion probably indicates a tendency to oligoclonality. In B6.Aec1/2, another mouse model of SjS, a clonal expansion with a tendency to oligoclonality in old mice was also observed (64). Thus, is important to study the B cell repertoire of the SjS patients to monitor whether or not a B cell clonal expansion is undergoing in order to foresee any lymphoma complications. One of the main striking characteristics of these clonally related IgG hybridomas is that all of them were polyreactive. In a very recent study of SjS patients it has also been shown a B cell clonal expansion in salivary gland of these subjects which they correlate to memory B cells. Although they observed a clonal expansion, they did not find either public clonotypes when analyzing the variable gene segment usage (65). In other autoimmune diseases such RA evidences have been found that indicate a B cell clonal expansion along with class-switching, SHM and autoreactivity, so the polyreactive expansion in SjS might be not that uncommon within autoimmune diseases (63).

The existence of clonally related B cells has already been found in follow up biopsies of the same SjS patient over an extended period of time (66). Moreover, we also identified that in the polyreactive antibody repertoire of NOD.H-2^h4^ mice the mutational degree in the VH region increases with mice aging, even though it is only a tendency, this mutational increment was not found in the B6 mice. In any case, the number of mutations observed in the polyreactive IgGs is very low, presenting 0-7 amino acid changes in the VH. This is in contrast with the polyreactive broadly neutralizing antibodies against HIV viral proteins, present in infected patients, which contained high levels of mutation indicating that they are derived from GC reactions (67). Moreover, almost no mutations were acquired in the CDR regions of these polyreactive antibodies displaying a near germline sequence. This is reminiscent of what has been observed in human patients with SLE, during acute flares, where polyclonally expanded autoantibody-secreting cells did not presented somatic hypermutations, consistent with a GC independent differentiation (68). In other autoimmune diseases autoantibodies develop due to failures of peripheral checkpoints after SHM in the GCs. For example, autoantibodies carrying high levels of somatic mutations in the CDR regions have been found in Pemphigus Vulgaris. These autoantibodies lost their binding capacity to their autoantigen when somatic mutations were reverted to the germline sequence, indicating that autoreactivity relies on somatic mutations (69).

In conclusion, our results indicate that in the NOD.H-2^h4^ mouse model of SjS, IgG B cells are mainly polyreactive and near germline. Moreover, these polyreactive IgG B cells might be expanding following an unknown antigen-driven positive selection process. Therefore, studying the possible pathogenicity of these polyreactive IgG2 antibodies and the contribution of MZ B cells to the generation of the autoimmune repertoire in the NOD.H-2^h4^ mouse model could help to understand better the pathological mechanism of SjS.

## Data Availability Statement

The original contributions presented in the study are included in the article/**Supplementary Material**. Further inquiries can be directed to the corresponding author.

## Ethics Statement

The animal study was reviewed and approved by Ethics Committee for Animal Experiments (CEEA) of the University of Barcelona. https://www.ub.edu/web/portal/en/research/research-at-the-ub/ethics-and-integrity/.

## Author Contributions

MSM: Investigation, Formal analysis, Visualization, Writing - Original Draft. RG-C: Investigation, Formal analysis. JP-O: Investigation, Formal analysis. MLRC: Investigation. JB: Writing - Review & Editing. JC: Writing - Review & Editing. PE: Supervision, Conceptualization, Writing - Original Draft, Writing - Review & Editing. All authors contributed to the article and approved the submitted version.

## Funding

This work was supported by the Ministerio de Economia y Competividad through Grant RTI2018-094440-B-I00 (to PE).

## Conflict of Interest

JB is CEO and cofounder and JC is CSO and cofounder of AlbaJuna Therapeutics.

The remaining authors declare that the research was conducted in the absence of any commercial or financial relationships that could be construed as a potential conflict of interest.

## References

[1] Cornec D Jamin C Pers JO. Sjögren’s Syndrome: Where do We Stand, and Where Shall We Go? J Autoimmun (2014) 51:109–14. 10.1016/j.jaut.2014.02.006 24612946

[2] Vitali C Bombardieri S Jonsson R Moutsopoulos HM Alexander EL Carsons SE. Classification Criteria for Sjögren’s Syndrome: A Revised Version of the European Criteria Proposed by the American-European Consensus Group. Ann Rheum Dis (2002) 61(6):554–8. 10.1136/ard.61.6.554 PMC1754137 12006334

[3] Mavragani CP Moutsopoulos HM. Sjögren Syndrome. CMAJ (2014) 186(15):E579–86. 10.1503/cmaj.122037 PMC4203623 24566651

[4] Tzioufas AG Tatouli IP Moutsopoulos HM. Autoantibodies in Sjögren’s Syndrome: Clinical Presentation and Regulatory Mechanisms. Presse Med (2012) 41(9 Pt 2):e451–60. 10.1016/j.lpm.2012.05.022 22840991

[5] Jonsson R Theander E Sjöström B Brokstad K Henriksson G. Autoantibodies Present Before Symptom Onset in Primary Sjögren Syndrome. JAMA (2013) 310(17):1854–5. 10.1001/jama.2013.278448 24193084

[6] Shen L Suresh L. Autoantibodies, Detection Methods and Panels for Diagnosis of Sjögren’s Syndrome. Clin Immunol (2017) 182:24–9. 10.1016/j.clim.2017.03.017 28390965

[7] Brito-Zerón P Baldini C Bootsma H Bowman SJ Jonsson R Mariette X. Sjögren Syndrome. Nat Rev Dis Primers (2016) 2:16047. 10.1038/nrdp.2016.47 27383445

[8] Roberts ME Kaminski D Jenks SA Maguire C Ching K Burbelo PD. Primary Sjögren’s Syndrome is Characterized by Distinct Phenotypic and Transcriptional Profiles of IgD+ Unswitched Memory B Cells. Arthritis Rheumatol (2014) 66(9):2558–69. 10.1002/art.38734 PMC4160119 24909310

[9] Mielle J Tison A Cornec D Le Pottier L Daien C Pers JO. B Cells in Sjögren’s Syndrome: From Pathophysiology to Therapeutic Target. Rheumatol (Oxford) (2019) key332. 10.1093/rheumatology/key332 30770916

[10] Youinou P Devauchelle-Pensec V Pers JO. Significance of B Cells and B Cell Clonality in Sjögren’s Syndrome. Arthritis Rheum (2010) 62(9):2605–10. 10.1002/art.27564 20496425

[11] Goules AV Tzioufas AG. Lymphomagenesis in Sjögren’s Syndrome: Predictive Biomarkers Towards Precision Medicine. Autoimmun Rev (2019) 18(2):137–43. 10.1016/j.autrev.2018.08.007 30572133

[12] Kapsogeorgou EK Voulgarelis M Tzioufas AG. Predictive Markers of Lymphomagenesis in Sjögren’s Syndrome: From Clinical Data to Molecular Stratification. J Autoimmun (2019) 104:102316. 10.1016/j.jaut.2019.102316 31431317

[13] Vivino FB Bunya VY Massaro-Giordano G Johr CR Giattino SL Schorpion A. Sjogren’s Syndrome: An Update on Disease Pathogenesis, Clinical Manifestations and Treatment. Clin Immunol (2019) 203:81–121. 10.1016/j.clim.2019.04.009 31022578

[14] Glauzy S Sng J Bannock JM Gottenberg JE Korganow AS Cacoub P. Defective Early B Cell Tolerance Checkpoints in Sjögren’s Syndrome Patients. Arthritis Rheumatol (2017) 69(11):2203–8. 10.1002/art.40215 PMC6062007 28704602

[15] Saadoun D Terrier B Bannock J Vazquez T Massad C Kang I. Expansion of Autoreactive Unresponsive CD21-/Low B Cells in Sjögren’s Syndrome-Associated Lymphoproliferation. Arthritis Rheum (2013) 65(4):1085–96. 10.1002/art.37828 PMC4479193 23279883

[16] Corsiero E Sutcliffe N Pitzalis C Bombardieri M. Accumulation of Self-Reactive Naïve and Memory B Cell Reveals Sequential Defects in B Cell Tolerance Checkpoints in Sjögren’s Syndrome. PloS One (2014) 9(12):e114575. 10.1371/journal.pone.0114575 25535746 PMC4275206

[17] Avrameas S Selmi C. Natural Autoantibodies in the Physiology and Pathophysiology of the Immune System. J Autoimmun (2013) 41:46–9. 10.1016/j.jaut.2013.01.006 23384670

[18] Avrameas S Alexopoulos H Moutsopoulos HM. Natural Autoantibodies: An Undersugn Hero of the Immune System and Autoimmune Disorders-a Point of View. Front Immunol (2018) 9:1320. 10.3389/fimmu.2018.01320 29946320 PMC6005843

[19] Dimitrov JD Planchais C Roumenina LT Vassilev TL Kaveri SV Lacroix-Desmazes S. Antibody Polyreactivity in Health and Disease: Statu Variabilis. J Immunol (2013) 191(3):993–9. 10.4049/jimmunol.1300880 23873158

[20] Meffre E O’Connor KC. Impaired B-cell Tolerance Checkpoints Promote the Development of Autoimmune Diseases and Pathogenic Autoantibodies. Immunol Rev (2019) 292(1):90–101. 10.1111/imr.12821 31721234 PMC9145185

[21] von Boehmer H Melchers F. Checkpoints in Lymphocyte Development and Autoimmune Disease. Nat Immunol (2010) 11(1):14–20. 10.1038/ni.1794 20016505

[22] Samuels J Ng YS Coupillaud C Paget D Meffre E. Impaired Early B Cell Tolerance in Patients With Rheumatoid Arthritis. J Exp Med (2005) 201(10):1659–67. 10.1084/jem.20042321 PMC2212916 15897279

[23] Yurasov S Wardemann H Hammersen J Tsuiji M Meffre E Pascual V. Defective B Cell Tolerance Checkpoints in Systemic Lupus Erythematosus. J Exp Med (2005) 201(5):703–11. 10.1084/jem.20042251 PMC2212839 15738055

[24] Dörner T Lipsky PE. Molecular Basis of Immunoglobulin Variable Region Gene Usage in Systemic Autoimmunity. Clin Exp Med (2005) 4(4):159–69. 10.1007/s10238-004-0051-2 15750762

[25] Karnell JL Mahmoud TI Herbst R Ettinger R. Discerning the Kinetics of Autoimmune Manifestations in a Model of Sjögren’s Syndrome. Mol Immunol (2014) 62(2):277–82. 10.1016/j.molimm.2014.05.006 24907930

[26] Braley-Mullen H Yu S. Nod.H-2h4 Mice: An Important and Underutilized Animal Model of Autoimmune Thyroiditis and Sjogren’s Syndrome. Adv Immunol (2015) 126:1–43. 10.1016/bs.ai.2014.11.001 25727287

[27] Romero X Cañete JD Engel P. Determination of Soluble Tumor Necrosis Factor Receptor 2 Produced by Alternative Splicing. Methods Mol Biol (2014) 1155:187–99. 10.1007/978-1-4939-0669-7_16 24788183

[28] Cossarizza A Chang HD Radbruch A Acs A Adam D Adam-Klages S. Guidelines for the Use of Flow Cytometry and Cell Sorting in Immunological Studies (Second Edition). Eur J Immunol (2019) 49(10):1457–973. 10.1002/eji.201970107 PMC7350392 31633216

[29] Carrillo J Puertas MC Planas R Pastor X Alba A Stratmann T. Anti-Peripherin B Lymphocytes are Positively Selected During Diabetogenesis. Mol Immunol (2008) 45(11):3152–62. 10.1016/j.molimm.2008.03.003 18433871

[30] Brochet X Lefranc MP Giudicelli V. Imgt/V-QUEST: The Highly Customized and Integrated System for IG and TR Standardized V-J and V-D-J Sequence Analysis. Nucleic Acids Res (2008) 1:W503–8. 10.1093/nar/gkn316 PMC2447746 18503082

[31] Giudicelli V Brochet X Lefranc MP. Imgt/V-Quest: IMGT Standardized Analysis of the Immunoglobulin (IG) and T Cell Receptor (TR) Nucleotide Sequences. Cold Spring Harb Protoc (2011) 2011(6):695–715. 10.1101/pdb.prot5633 21632778

[32] Ivanov II Schelonka RL Zhuang Y Gartland GL Zemlin M Schroeder HW Jr. Development of the Expressed Ig Cdr-H3 Repertoire is Marked by Focusing of Constraints in Length, Amino Acid Use, and Charge That are First Established in Early B Cell Progenitors. J Immunol (2005) 174(12):7773–80. 10.4049/jimmunol.174.12.7773 15944280

[33] Mietzner B Tsuiji M Scheid J Velinzon K Tiller T Abraham K. Autoreactive IgG Memory Antibodies in Patients With Systemic Lupus Erythematosus Arise From Nonreactive and Polyreactive Precursors. Proc Natl Acad Sci USA (2008) 105(28):9727–32. 10.1073/pnas.0803644105 PMC2474524 18621685

[34] Heimbächer C Hansen A Pruss A Jacobi A Reiter K Lipsky PE. Immunoglobulin Vkappa Light Chain Gene Analysis in Patients With Sjögren’s Syndrome. Arthritis Rheum (2001) 44(3):626–37. 10.1002/1529-0131(200103)44:3<626::AID-ANR111>3.0.CO;2-T 11263777

[35] Tiller T Kofer J Kreschel C Busse CE Riebel S. Development of Self-Reactive Germinal Center B Cells and Plasma Cells in Autoimmune Fc gammaRIIB-deficient Mice. J Exp Med (2010) 207(12):2767–78. 10.1084/jem.20100171 PMC2989760 21078890

[36] Mouquet H Nussenzweig MC. Polyreactive Antibodies in Adaptive Immune Responses to Viruses. Cell Mol Life Sci (2012) 69(9):1435–45. 10.1007/s00018-011-0872-6 PMC11114792 22045557

[37] Gunti S Kampylafka EI Tzioufas AG Notkins AL. Polyreactive Antibodies in the Circulation of Patients With Systemic Lupus Erythematosus. Lupus (2015) 24(14):1567–9. 10.1177/0961203315603144 PMC4651727 26385217

[38] Gunti S Notkins AL. Polyreactive Antibodies: Function and Quantification. J Infect Dis (2015) 212 Suppl 1(Suppl 1):S42–6. 10.1093/infdis/jiu512 PMC4490204 26116731

[39] Argyropoulou OD Gunti S Kapsogeorgou EK Notkins AL Tzioufas AG. Decrease in the Ratio of Polyreactive IgG Titers With IgG Concentration is Associated With Long-Term Complications of Primary Sjögren’s Syndrome. Clin Exp Rheumatol (2018) 36 Suppl 112(3):239–40. 30156547

[40] Wang X Xia Y. Anti-Double Stranded Dna Antibodies: Origin, Pathogenicity, and Targeted Therapies. Front Immunol (2019) 10:1667. 10.3389/fimmu.2019.01667 31379858 PMC6650533

[41] Ehlers M Fukuyama H McGaha TL Aderem A Ravetch JV. Tlr9/MyD88 Signaling is Required for Class Switching to Pathogenic IgG2a and 2b Autoantibodies in SLE. J Exp Med (2006) 203(3):553–61. 10.1084/jem.20052438 PMC2118244 16492804

[42] Groom JR Fletcher CA Walters SN Grey ST Watt SV Sweet MJ. BAFF and MyD88 Signals Promote a Lupuslike Disease Independent of T Cells. J Exp Med (2007) 204(8):1959–71. 10.1084/jem.20062567 PMC2118661 17664289

[43] Puñet-Ortiz J Sáez Moya M Cuenca M Caleiras E Lazaro A Engel P. Ly9 (CD229) Antibody Targeting Depletes Marginal Zone and Germinal Center B Cells in Lymphoid Tissues and Reduces Salivary Gland Inflammation in a Mouse Model of Sjögren’s Syndrome. Front Immunol (2018) 9:2661. 10.3389/fimmu.2018.02661 30519241 PMC6251324

[44] Zouali M Richard Y. Marginal Zone B-cells, a Gatekeeper of Innate Immunity. Front Immunol (2011) 2:63. 10.3389/fimmu.2011.00063 22566852 PMC3341996

[45] Rolf J Motta V Duarte N Lundholm M Berntman E. The Enlarged Population of Marginal Zone/CD1d(High) B Lymphocytes in Nonobese Diabetic Mice Maps to Diabetes Susceptibility Region Idd11. J Immunol (2005) 174(8):4821–7. 10.4049/jimmunol.174.8.4821 15814708

[46] Stolp J Mariño E Batten M Sierro F Cox SL. Intrinsic Molecular Factors Cause Aberrant Expansion of the Splenic Marginal Zone B Cell Population in Nonobese Diabetic Mice. J Immunol (2013) 191(1):97–109. 10.4049/jimmunol.1203252 23740954

[47] Mackay F Woodcock SA Lawton P Ambrose C Baetscher M. Mice Transgenic for BAFF Develop Lymphocytic Disorders Along With Autoimmune Manifestations. J Exp Med19 (1999) 190(11):1697–710. 10.1084/jem.190.11.1697 PMC2195729 10587360

[48] Groom J Kalled SL Cutler AH Olson C Woodcock SA. Association of BAFF/BLyS Overexpression and Altered B Cell Differentiation With Sjögren’s Syndrome. J Clin Invest (2002) 109(1):59–68. 10.1172/JCI14121 11781351 PMC150825

[49] Berland R Fernandez L Kari E Han JH Lomakin I Akira S. Toll-Like Receptor 7-Dependent Loss of B Cell Tolerance in Pathogenic Autoantibody Knockin Mice. Immunity (2006) 25(3):429–40. 10.1016/j.immuni.2006.07.014 16973388

[50] Umiker BR McDonald G Larbi A Medina CO Hobeika E Reth M. Production of IgG Autoantibody Requires Expression of Activation-Induced Deaminase in Early-Developing B Cells in a Mouse Model of SLE. Eur J Immunol (2014) 44(10):3093–108. 10.1002/eji.201344282 PMC4197127 25044405

[51] Giltiay NV Chappell CP Sun X Kolhatkar N Teal TH Wiedeman AE. Overexpression of TLR7 Promotes Cell-Intrinsic Expansion and Autoantibody Production by Transitional T1 B Cells. J Exp Med (2013) 210(12):2773–89. 10.1084/jem.20122798 PMC3832927 24145511

[52] Liao W Hua Z Liu C Lin L Chen R Hou B. Characterization of T-Dependent and T-Independent B Cell Responses to a Virus-like Particle. J Immunol (2017) 198(10):3846–56. 10.4049/jimmunol.1601852 28416599

[53] Harley JB Alexander EL Bias WB Fox OF Provost TT Reichlin M. Anti-Ro (Ss-A) and anti-La (Ss-B) in Patients With Sjögren’s Syndrome. Arthritis Rheum (1986) 29(2):196–206. 10.1002/art.1780290207 3485431

[54] Garberg H Jonsson R Brokstad KA. The Serological Pattern of Autoantibodies to the Ro52, Ro60, and La48 Autoantigens in Primary Sjögren’s Syndrome Patients and Healthy Controls. Scand J Rheumatol (2005) 34(1):49–55. 10.1080/03009740510017940 15903026

[55] Lindop R Arentz G Thurgood LA Reed JH Jackson MW Gordon TP. Pathogenicity and Proteomic Signatures of Autoantibodies to Ro and La. Immunol Cell Biol (2012) 90(3):304–9. 10.1038/icb.2011.108 22249199

[56] Deshmukh US Bagavant H Fu SM. Role of anti-DNA Antibodies in the Pathogenesis of Lupus Nephritis. Autoimmun Rev (2006) 5(6):414–8. 10.1016/j.autrev.2005.10.010 16890896

[57] Madaio MP Carlson J Cataldo J Ucci A Migliorini P Pankewycz O. Murine Monoclonal anti-DNA Antibodies Bind Directly to Glomerular Antigens and Form Immune Deposits. J Immunol (1987) 138(9):2883–9. 3553329

[58] Pankewycz OG Migliorini P Madaio MP. Polyreactive Autoantibodies are Nephritogenic in Murine Lupus Nephritis. J Immunol (1987) 139(10):3287–94. 3500215

[59] Zhang J Jacobi AM Wang T Berlin R Volpe BT Diamond B. Polyreactive Autoantibodies in Systemic Lupus Erythematosus Have Pathogenic Potential. J Autoimmun (2009) 33(3-4):270–4. 10.1016/j.jaut.2009.03.011 PMC2783480 19398190

[60] Kowal C Degiorgio LA Lee JY Edgar MA Huerta PT Volpe BT. Human Lupus Autoantibodies Against NMDA Receptors Mediate Cognitive Impairment. Proc Natl Acad Sci USA (2006) 103(52):19854–9. 10.1073/pnas.0608397104 PMC1702320 17170137

[61] Al Kindi MA Colella AD Chataway TK Jackson MW Wang JJ Gordon TP. Secreted Autoantibody Repertoires in Sjögren’s Syndrome and Systemic Lupus Erythematosus: A Proteomic Approach. Autoimmun Rev (2016) 15(4):405–10. 10.1016/j.autrev.2016.01.008 26804757

[62] Zuckerman NS Hazanov H Barak M Edelman H Hess S Shcolnik H. Somatic Hypermutation and Antigen-Driven Selection of B Cells are Altered in Autoimmune Diseases. J Autoimmun (2010) 35(4):325–35. 10.1016/j.jaut.2010.07.004 20727711

[63] Bashford-Rogers R Smith K Thomas DC. Antibody Repertoire Analysis in Polygenic Autoimmune Diseases. Immunology (2018) 155(1):3–17. 10.1111/imm.12927 29574826 PMC6099162

[64] Meng W Li Y Xue E Satoh M Peck AB. B-Cell Tolerance Defects in the B6.Aec1/2 Mouse Model of Sjögren’s Syndrome. J Clin Immunol (2012) 32(3):551–64. 10.1007/s10875-012-9663-6 PMC3351565 22350147

[65] Visser A Verstappen GM van der Vegt B Vissink A Bende RJ Bootsma H. Repertoire Analysis of B-Cells Located in Striated Ducts of Salivary Glands of Patients With Sjögren’s Syndrome. Front Immunol (2020) 11:1486. 10.3389/fimmu.2020.01486 32760405 PMC7372116

[66] Bahler DW Swerdlow SH. Clonal Salivary Gland Infiltrates Associated With Myoepithelial Sialadenitis (Sjögren’s Syndrome) Begin as Nonmalignant Antigen-Selected Expansions. Blood (1998) 91(6):1864–72. 10.1182/blood.V91.6.1864 9490668

[67] Haynes BF Burton DR Mascola JR. Multiple Roles for HIV Broadly Neutralizing Antibodies. Sci Transl Med (2019) 11(516):eaaz2686. 10.1126/scitranslmed.aaz2686 31666399 PMC7171597

[68] Tipton CM Fucile CF Darce J Chida A Ichikawa T. Diversity, Cellular Origin and Autoreactivity of Antibody-Secreting Cell Population Expansions in Acute Systemic Lupus Erythematosus. Nat Immunol (2015) 16: (7):755–65. 10.1038/ni.3175 PMC4512288 26006014

[69] Di Zenzo G Di Lullo G Corti D Calabresi V Sinistro A Vanzetta F. Pemphigus Autoantibodies Generated Through Somatic Mutations Target the Desmoglein-3 Cis-Interface. J Clin Invest (2012) 122(10):3781–90. 10.1172/JCI64413 PMC3461925 22996451

