# Supplementary material for: Autoimmune B Cell Repertoire in a Mouse Model of Sjögren’s Syndrome
Source: Front Immunol. 2021 Apr 23;12:666545. doi: 10.3389/fimmu.2021.666545 (PMC8103202; doi:10.3389/fimmu.2021.666545)
Supplement: Supplementary file 1 [file DataSheet_1.docx]

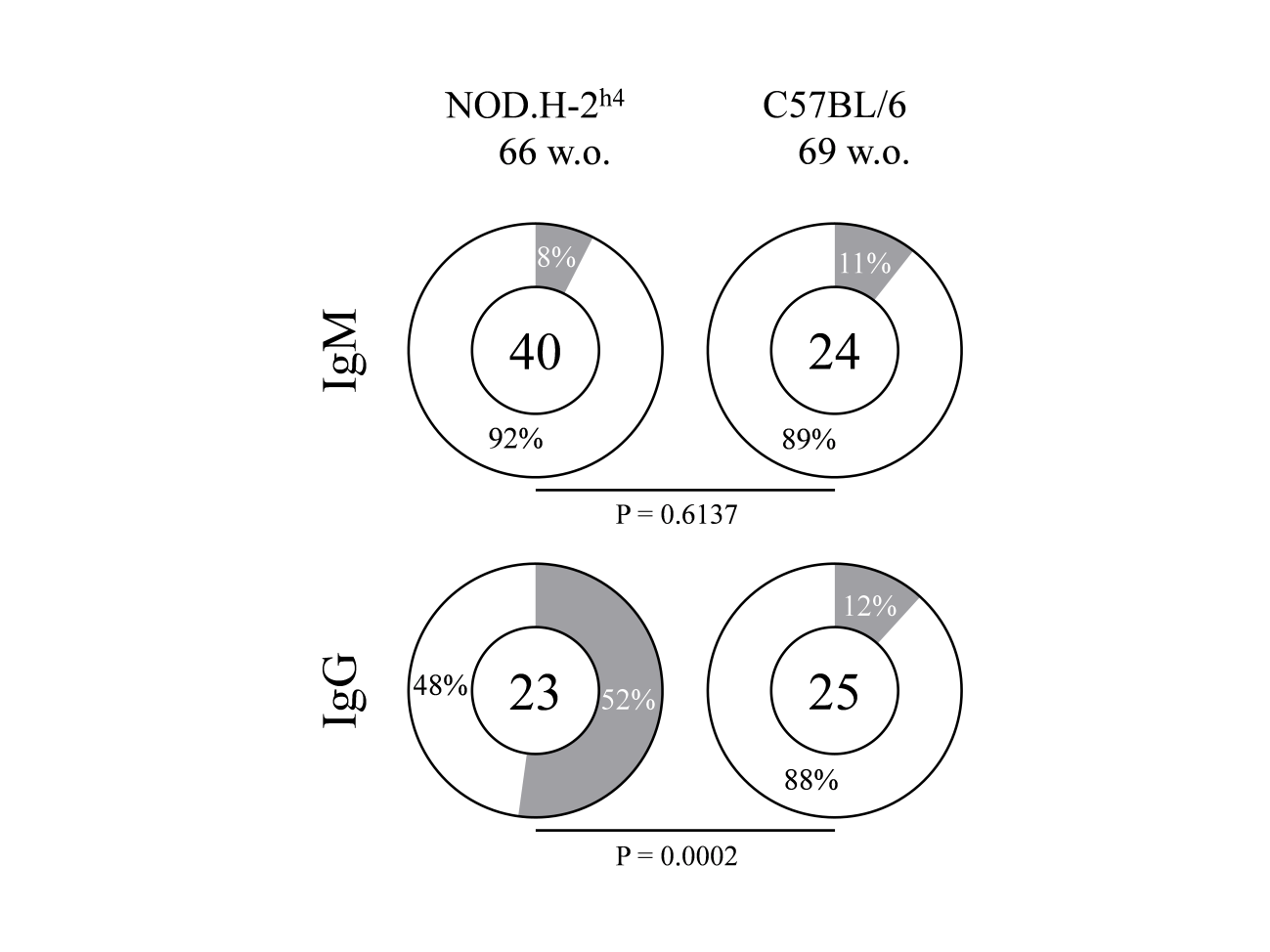


Supplementary Figure 1. Frequency of dsDNA specific antibodies. Pie charts showing the proportion of dsDNA reactive (light grey) and non-reactive (white) antibodies for NOD.H-2^h4^ and C57BL/6J, determined by ELISA Data for each age NOD.H-2^h4^: 66 w.o., C57BL/6J: 69 w.o.; and both IgM and IgG isotypes are shown. The number of tested antibodies is indicated in the pie chart center. Data are pooled from independent experiments for each mouse age. Pie charts were analyzed with GraphPad Prism 6 and P-values were calculated by χ2 test.

Supplementary Table 1. List of used VH primers.

| **Chain** | **Position** | **Name** | **Sequence (5’-3’)** |
| --- | --- | --- | --- |
| **VH** | AS | Cγ1-outer | GGAAGGTGTGCACACCGCTGGAC |
|  | AS | IgM-1 | GGAAGACATTTGGGAAGGAC |
|  | S | MS VHE | GGGAATTCGAGGTGCAGCTGCAGGAGTCTG |
|  | S | MHALT1 | ATGGRATGSAGCTGKGTMATSCTCTT |
|  | S | MHALT2 | ATGRACTTCGGGYTGAGCTKGGTTTT |
|  | S | MHALT3 | ATGGCTGTCTTGGGGCTGCTCTTCT |
|  | S | MHALT4 | CACCATGGRCAGRCTTACWTYY |
|  | S | AgeIP-mVH02 | CTGCAACCGGTGTACATTCCCAGGTGCAGCTGCAGCAGTCTGG |
|  | S | AgeIP-mVH13 | CTGCAACCGGTGTACATTCCCAGGTTCAGCTGCAACAGTCTGA |
|  | S | AgeIP-mVH20 | CTGCAACCGGTGTACATTCCCAGGTGCAGCTTGTAGAGACCGG |
|  | S | AgeIP-mVH21 | CTGCAACCGGTGTACATTCCCAGATGCAGCTTCAGGAGTCAGG |

Supplementary Table 2. List of used VL primers.

| **Chain** | **Position** | **Name** | **Sequence (5’-3’)** |
| --- | --- | --- | --- |
| **VL** | AS | mCK | GATGGTGGGAAGATGGATACAGTT |
|  | S | MLALT1 | ATGGAGACAGACACACTCCTGCTAT |
|  | S | MLALT2 | ATGGATTTTCAGGTGCAGATTTTCAG |
|  | S | MLALT3 | ATGRAGTCACAKACYCAGGTCTTYRTA |
|  | S | MLALT4 | ATGAGGKCCCCWGCTCAGYTYCTKGGR |
|  | S | MLALT5 | ATGAAGTTGCCTGTTAGGCTGTTG |
|  | S | MLALT6 | ATGATGAGTCCTGCCCAGTTCC |
|  | S | AgeIP-mVK14 | CTGCAACCGGTGTACATTCCGATATCCAGATGACACAGACTACA |
|  | S | AgeIP-mVK17 | CTGCAACCGGTGTACATTCCGACATCCAGATGACACAATCTTCA |
|  | S | AgeIP-mVK22 | CTGCAACCGGTGTACATTCCAACATTGTAATGACCCAATCTCCC |
|  | S | AgeIP-mVK24 | CTGCAACCGGTGTACATTCCGATATTGTGATGACTCAGGCTGCA |

Supplementary Table 3 IGHV and IGKV of IgM antibodies derived from aging C57BL/6J mice (26, 47 and 69 w.o)

|  |  | **Heavy Chain** | | | | | **Light Chain** | | | |
| --- | --- | --- | --- | --- | --- | --- | --- | --- | --- | --- |
|  | **Hybridoma ID** | **IGHV** | **IGHD** | **IGHJ** | **CDR3** | **Length** | **IGκV** | **IGκJ** | **CDR3** | **Length** |
| **26**  **w.o.** | **B6.1.15** | 1-72 | 1-1 | 3 | ARPNYSGNSLAY | 12 | 3-10 | 1 | QQNNEDPWT | 9 |
|  | **B6.1.33** | 1-64 | 1-1 | 2 | ARYFPITTVHFDY | 13 | 1-117 | 1 | FQGSHVPWT | 9 |
|  | **B6.1.34** | 1-72 | 1-1 | 1 | AYRYFDV | 7 | 4-59 | 5 | QQWSSNPLT | 9 |
|  | **B6.1.36** | 1-26 | - | 3 | ASWFAY | 6 | 15-103 | 1 | QQGQSYPWT | 9 |
|  | **B6.1.89** | 1-5 | - | 3 | TSPFAY | 6 | 3-5 | 2 | QQSNEDPYT | 9 |
|  |  |  |  |  |  |  |  |  |  |  |
| **47**  **w.o.** | **B6.3.64** |  |  |  | NS |  | 12-41 | 1 | QHFWSTPPT | 9 |
|  |  |  |  |  |  |  |  |  |  |  |
| **69**  **w.o.** | **B6.2.93** | 8-8 | 1-1 | 3 | ARTYYYGTSYWYFDV | 15 | 4-74 | 1 | HQYHRSPRT | 9 |

IGHV, Immunoglobulin heavy chain variable region gene; IGHD, Immunoglobulin heavy chain diversity region gene; IGHJ, Immunoglobulin heavy chain joining region gene; IGκV, Immunoglobulin kappa chain variable region gene; IGκJ, Immunoglobulin kappa chain joining region gene.

Supplementary Table 4 IGHV and IGKV of IgG antibodies derived from aging C57BL/6J mice (26, 47 and 69 w.o)

|  |  |  | **Heavy Chain** | | | | | **Light Chain** | | | |
| --- | --- | --- | --- | --- | --- | --- | --- | --- | --- | --- | --- |
|  | **Hybridoma ID** | **IgG Subclass** | **IGHV** | **IGHD** | **IGHJ** | **CDR3** | **Length** | **IGκV** | **IGκJ** | **CDR3** | **Length** |
| **26 w.o.** | **B6.1.7** | IgG3 | 1-26 | 1-1 | 1 | VRGSHWYFDV | 10 | 12-46 | 5 | QHFWGTPLT | 9 |
|  |  |  |  |  |  |  |  |  |  |  |  |
| **47 w.o.** | **B6.3.9** | IgG2c | 1-80 | 1-1 | 3 | AREGVLLPLFAY | 12 | 1-110 | 2 | SQSTHVPYT | 9 |
|  |  |  |  |  |  |  |  |  |  |  |  |
| **69 w.o.** | **B6.2.26** | IgG2b | 3-1 | 1-1 | 4 | ARGGNYDGYAMDY | 13 | 12-89 | 2 | QNVLSTPYT | 9 |
|  | **B6.2.58** | IgG3 | 1-80 | 1-1 | 2 | ATSYYFGSNYYFDY | 14 | 10-96 | 2 | QQGKTLPYT | 9 |
|  | **B6.2.74** | IgG3 | 1-80 | 1-1 | 2 | ATSYYFGTNYYFDY | 14 | 14-111 | 2 | LQYDEHPYT | 9 |
|  | **B6.2.101** | IgG3 | 1-80 | 1-1 | 2 | ATSYYFGSNYYFDY | 14 | 10-96 | 2 | QQGNTVPYT | 9 |
|  | **B6.2.140** | IgG2b | 3-6 | 1-1 | 2 | ARITTVPFDY | 10 | 10-96 | 2 | QQGKTLPYT | 9 |
|  | **B6.2.142** | IgG2b | 1-19 | 1-1 | 3 | ARRKGSSAPWFAY | 13 | 12-46 | 1 | QHFWGTPRT | 9 |
|  | **B6.2.166** | IgG3 | 1-50 | 2-5 | 1 | AIYSKSNYFDV | 11 | 4-74 | 1 | HQYHRSPRT | 9 |

IGHV, Immunoglobulin heavy chain variable region gene; IGHD, Immunoglobulin heavy chain diversity region gene; IGHJ, Immunoglobulin heavy chain joining region gene; IGκV, Immunoglobulin kappa chain variable region gene; IGκJ, Immunoglobulin kappa chain joining region gene.

Supplementary Table 5. VH & VK mutations and localization of the two sets of clonally related antibodies.

|  | Hybridoma ID | **FR1** | **CDR1** | **FR2** | **CDR2** | **FR3** | **CDR3** |
| --- | --- | --- | --- | --- | --- | --- | --- |
| Heavy Chain | H2h4.7.21 | V5›Q | − | − | − | Y88›C/A100›G/  M101›I | − |
|  | H2h4.7.25 | V5›Q | − | − | − | Y88›C/A100›G/  M101›I | − |
|  | H2h4.7.8 | Q6›E | − | − | − | − | − |
|  | H2h4.7.50 | Q6›E | − | − | − | − | − |
|  | H2h4.7.94 | Q6›E | − | Y55›H | G63›A | A87›T | − |
|  |  |  |  |  |  |  |  |
| Light Chain | H2h4.7.21 | V13›A | Y36›D/  N38›Y | − | − | N66›L/S93›R/  F99›V/G100›A/  S101›R | F107›Y/W108›Y/  G109›S |
|  | H2h4.7.25 | V13›A | Y36›D/  N38›Y | − | − | N66›L/S93›R/  F99›V/G100›A/  S101›R | F107›Y/W108›Y/  G109›S |
|  | H2h4.7.8 | I19›V | − | S49›P | − | D98›G/V99›A | Q106›H/I110›L/  P111›L |
|  | H2h4.7.50 | I19›V | − | S49›P | − | D98›G/V99›A | Q106›H/I110›L/  P111›L |
|  | H2h4.7.94 | M11›L/  I19›V | N37›S | S49›P | − | D98›G/V99›A | Q106›H/I110›L/  P111›L |
